# Strengthening health technology assessment systems in the global south: a comparative analysis of the HTA journeys of China, India and South Africa

**DOI:** 10.1080/16549716.2018.1527556

**Published:** 2018-10-17

**Authors:** Kim MacQuilkan, Peter Baker, Laura Downey, Francis Ruiz, Kalipso Chalkidou, Shankar Prinja, Kun Zhao, Thomas Wilkinson, Amanda Glassman, Karen Hofman

**Affiliations:** a Priority Cost Effective Lessons for System Strengthening South Africa (PRICELESS SA), Faculty of Health Sciences, School of Public Health, University of Witwatersrand, Johannesburg, South Africa; b Global Health and Development Group, Institute of Global Health Innovation, Imperial College London, London, UK; c School of Public Health, Post Graduate Institute of Medical Education and Research (PGIMER), Chandigarh, India; d Division of Health Technology Assessment and Policy Evaluation, China National Health Development Research Center (CHNHDR), Ministry of Health, Beijing, China; e Center for Global Development, Washington, DC, USA

**Keywords:** Capacity building, south-south collaboration, LMICs, priority-setting, HTA

## Abstract

**Background**: Resource allocation in health is universally challenging, but especially so in resource-constrained contexts in the Global South. Pursuing a strategy of evidence-based decision-making and using tools such as Health Technology Assessment (HTA), can help address issues relating to both affordability and equity when allocating resources. Three BRICS and Global South countries, China, India and South Africa have committed to strengthening HTA capacity and developing their domestic HTA systems, with the goal of getting evidence translated into policy. Through assessing and comparing the HTA journey of each country it may be possible to identify common problems and shareable insights.

**Objectives**: This collaborative paper aimed to share knowledge on strengthening HTA systems to enable enhanced evidence-based decision-making in the Global South by: Identifying common barriers and enablers in three BRICS countries in the Global South; and Exploring how South-South collaboration can strengthen HTA capacity and utilisation for better healthcare decision-making.

**Methods**: A descriptive and explorative comparative analysis was conducted comprising a Within-Case analysis to produce a narrative of the HTA journey in each country and an Across-Case analysis to explore both knowledge that could be shared and any potential knowledge gaps.

**Results**: Analyses revealed that China, India and South Africa share many barriers to strengthening and developing HTA systems such as: (1) Minimal HTA expertise; (2) Weak health data infrastructure; (3) Rising healthcare costs; (4) Fragmented healthcare systems; and (5) Significant growth in non-communicable diseases. Stakeholder engagement and institutionalisation of HTA were identified as two conducive factors for strengthening HTA systems.

**Conclusion**: China, India and South Africa have all committed to establishing robust HTA systems to inform evidence-based priority setting and have experienced similar challenges. Engagement among countries of the Global South can provide a supportive platform to share knowledge that is more applicable and pragmatic.

## Background

Low- and Middle-Income Countries (LMICs) face difficult decisions regarding resource allocation, due to scarce resources and large disease burdens [1,2]. Lack of systematic priority setting can lead to inefficient resource allocation and poor-quality healthcare [3]. In light of this and the global trend towards pursuing Universal Health Coverage (UHC), countries are increasingly acknowledging the importance of explicit priority setting and evidence-based decision-making [4,5]. Priority-setting tools, for example Health Technology Assessments (HTAs), provide valuable evidence which could be useful for decision-making in LMICs, both in terms of maximising health and enhancing equity [4,6,7].

In light of this, many LMICs have begun taking initial steps towards institutionalising HTA. Developing a formal and robust HTA system, where there are clear links between evidence generation and its application to policy, can be difficult to achieve in unsupportive political environments and limited resources, challenges that are particularly marked in LMICs. In addition the wider healthcare system and country setting should be carefully considered [8–10]. Despite the context-specific nature of establishing and strengthening HTA processes, there are several potential lessons to be shared across countries of similar income and development levels, such as Global South and BRICS countries [10], several of which are on a path towards UHC [9]. Sharing knowledge through South-South collaboration and capacity building amongst these countries could provide valuable insights and support. South-South collaboration in health developed from the more politically orientated concept of South-South cooperation (SCC), which arose from the Bandung Conference in 1955. The concept of SSC and resulting term ‘Global South’ was developed to replace disempowering terms such as, ‘third-world’ or ‘developing’ countries [11].

Due to large variation between countries within the Global South, knowledge sharing for HTA would be more useful between countries which share other relevant factors. For instance, the BRICS (Brazil, Russia, India, China, and South Africa) countries are all on the path to UHC and have started developing HTA systems [9,12]. In addition, China, India and South Africa, are represented in a global network, the International Decision Support Initiative (iDSI) which aims to strengthen capacity to undertake HTA and support the utilisation of HTA evidence for resource allocation decisions in LMICs. The iDSI Theory of Change is based on impact generating and practical partnerships to strengthen country decision making institutions, with the ultimate goal of achieving better health through the adoption of policies based in evidence [11,12]. As such, an integral part of the network is knowledge sharing, collaboration and capacity building [13–15]. Knowledge sharing is vital to providing potential insights, aids and strategies for strengthening evidence-based decision making and the employment of tools such as HTA [4,16]. An exploration of the development of HTA systems in China, India and South Africa could provide beneficial insights for the other BRICS countries Brazil and Russia, as well as other Global South countries. Due to the context-specific nature of HTA system development, the derived knowledge should aim to be detailed yet pragmatic.

This collaborative paper aims to share knowledge on strengthening HTA capacity and systems to enable enhanced evidence-based decision-making by:

1. Providing a rich description of the HTA Journey in China, India and South Africa;
2. Comparing HTA systems and development across the countries to produce knowledge that can be shared (barriers and enablers); and
3. Identifying how South-South collaboration can strengthen HTA capacity and utilisation of HTA for better healthcare decision-making.

## Methods

The analytic process adopted was a case-orientated approach to comparative analysis. The specific case-orientated approach was most appropriate for the study to elicit context-rich information across many variables, from a few case countries, and involved both within-case and across-case analyses [17–19]. Data collection and analysis was conducted iteratively, involving national and international authors, including those directly involved in the development of HTA in the three countries covered by this paper.

### Case selection

China, India and South Africa were purposively selected as country cases as they are all LMICs in the Global South, on the path towards UHC, that are aiming to strengthen HTA structures and related processes. These three selected countries are also part of BRICS and represented on the iDSI network, which facilitated a collaborative approach in developing this paper. A collaborative approach was an essential component in the selection of these countries in order to identify and incorporate as much relevant information as possible and facilitate shared learning in the process.

### Data collection

Three main data sources were utilised iteratively:

- Compilation of a standardised table by an iDSI partner working on a related project in each country (see below – Framework);
- A systematic search of literature (see below – Systematic search); and
- Materials from workshop proceedings of an iDSI South-South Knowledge Sharing Workshop on HTA, held in South Africa, November 2016 (see below – Workshop proceedings [14].

The use of the three different data sources, in addition to data verification by key reviewers, provided for the triangulation of information and subsequently a complete overview of each country. The ENTREQ statement was utilised to guide data collection and synthesis [20].

#### Framework

In the first instance, a standardised table derived from the categories found in the Global Survey on Health Technology Assessment by National Authorities [8], was completed by an iDSI partner and/or associate with experience working in each case country. Categories were then amended and/or additional categories added to the table where necessary, based on the literature review and a review of workshop materials, resulting in seven main categories (see Table 1 below). The amended table was utilised to guide data extraction from the systematic literature search and materials derived from the workshop proceedings (see below – data extraction and data analysis)

**Table 1.** Amended data extraction framework derived from the WHO global health survey on health technology assessment by national authorities [8].

Category	Sub-Category
1. Utilization of HTA in public sector decision-making	Formal ‘information-gathering process’ for decision making; Legislative requirements for considering HTA findings; Purposes of undertaking HTA; Types of technologies or interventions assessed
2. Scope of HTA and availability of guidelines	Aspects considered in HTA; Guidelines for developing HTA
3. Institutional capacity and human resources supporting HTA	National HTA organization; Number of staff members in HTA organizations; Requests for HTAs; Professionals involved in HTA preparation and decision making
4. Governance of HTA process	Conflict of interest declaration; Communicating the outcomes of HTA; HTA entity impact on policy and decision making; Stakeholder engagement
5. Requirements for strengthening HTA capacity	Main barrier for producing HTA and using HTA findings in decision making; *Enablers in the progress towards institutionalisation of HTA Academic or training programmes to support capacity building for HTA
*6. Barriers to Institutionalising HTA	No sub-categories
*7. Future goals for HTA system development	No sub-categories

* Additional categories (6 & 7) and sub-category (within category 5) added to WHO framework during framework amendment – see Framework above.

[8] World Health Organization. Global Survey on Health Technology Assessment by National Authorities. [Internet]. Geneva: World Health Organization; 2015. Available from: http://www.who.int/health-technology-assessment/MD_HTA_oct2015_final_web2.pdf.

**Table 2.** Common problems, conducive factors and recommendations for HTA development in Asia [10].

Problems	Conducive Factors	Recommendations
Silo-based decision-making Low-quality decision-making criteria Strict control on research dissemination Respect for expert (senior) opinions or authorities is held in higher than evidence-based research	A high proportion of public investment and strategic purchasing mechanism Political will, leadership, and legislation A good health information infrastructure Local training on HTA-related disciplines Effective collaboration between HTA agencies/programs and local stakeholders Settings’ independence from external support or international aid	Human resource development Core team or HTA institutes Linking HTA to policy decision-making mechanisms HTA legislation International collaboration

#### Systematic Search of Literature

**Figure 1.**
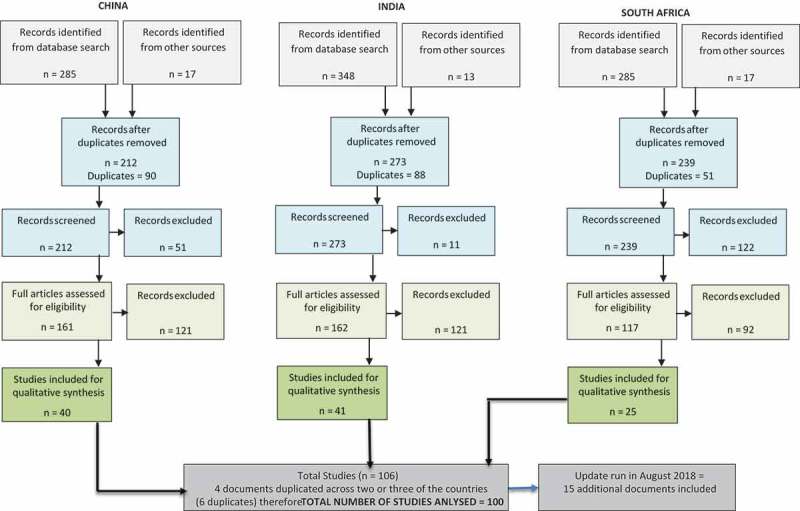
Systematic search process.

Second, a systematic search of literature for documents related to priority-setting and HTA in China, India and South Africa was conducted in the PubMed and Scopus databases in September 2017 and an update run in August 2018. Filters were utilised, including limiting papers to those published in English language. Figure 1 shows the systematic search process (See S1 – Search strategy for further detail on search terms, screening and eligibility criteria).

#### Workshop proceedings

The materials from the workshop proceedings of the iDSI workshop were included, as this meeting brought together stakeholders from China, India, and South Africa as well as Indonesia, Cambodia, Thailand and the UK to facilitate knowledge sharing on HTA system strengthening, through North-South and South-South Collaboration. The workshop involved context-specific presentations from each participant country and many in-depth discussions. Rich and context-specific issues were highlighted along with common challenges and goals [14].

### Data extraction, appraisal and analysis

Data was extracted from the included documents utilising the amended framework detailed above (see Methodology – Data Collection – Framework). The aim of the paper was to provide comprehensive and rich descriptions thus data extraction was guided by the amended framework and where there were multiple sources of information, the most up-to-date document of best quality was utilised. Quality assessment was guided by the CASP Qualitative checklist [21]. The completed framework guided the analysis of data for each country (Within-Case Analysis) to produce a narrative of the HTA journey of each country. In addition, information was compared across each country for every sub-category of the framework (Cross-Case Analysis). Results of both analyses were further examined inductively for common themes.

## Findings

The results of the two analyses are presented below (Full data extraction is provided as supplementary material – See S2).

### Within-case analysis – HTA journey

#### China’s HTA journey

HTA has been a topic of interest in China for over three decades. The use of evidence-based priority setting is supported by academics and the government, yet HTA is still to be utilised comprehensively in decision-making [16,22–25]. A challenge to standard utilisation of HTA, in a coordinated approach, is the fragmented health system [26–28]. Although a large percentage of the population (97%) is covered by three different health insurance arrangements, the schemes are funded and managed separately. In addition, funding is pooled at various government levels (county or municipal) for each scheme. Subsequently there are different reimbursement schedules and rates for each scheme and in some cases for each level, contributing to an inequality and inequity within the healthcare system. China aims to consolidate the schemes by 2020 and have one single payer, under the Ministry of Human Resources and Social Security (MOHRSS) [26,27,29–36].

There has also been recent political commitment for the coordination of HTA at a national level. In 2016, a national policy document entitled, ‘Guidance on Strengthening Scientific and Technological Outcomes Transformation in Healthcare area’ was issued by five ministries. The policy enables the formation of a HTA system and designates responsibility for national HTA to the NHFPC (National Health and Family Planning Commission), further demonstrating government’s commitment to strengthening HTA in China [16,37]. The document details several actions including the establishment of national HTA centres. In line with this approach, the China National Health Development Research Center (CNHDRC) under the governance of NHFPC launched an evidence network to support HTA. The network will provide a framework for pooling and integrating all HTA resources and relevant stakeholders in China, under the coordination of CNHDRC. In addition to having a pivotal role in HTA institutionalisation in China, the network seeks to engage internationally, sharing experiences and skills within the framework of ‘One Belt and One Road’ [38–41]. Earlier this year, China adopted the National Health Commission which replaces the NHFPC and aims to incorporate HTA more systematically into decision-making [42,43].

#### India’s HTA journey

Previously, explicit priority setting or HTA has not been systematically incorporated into decision-making in India for resource allocation or reimbursement [44–47]. This can be difficult in the context of the current healthcare system, which is complex and fragmented with several different insurance and ‘assurance’ arrangements, at both the central and state level. As of 2014, less than 20% of the population were covered by government schemes with majority of the population utilising private health care providers. Out-of-Pocket Payments (OOPs) accounted for 62% of total health expenditure [48], leaving millions of the population at risk of falling into debt or poverty [46,49–52]. Government expenditure on healthcare has been low (1.04% of GDP in 2014) and the rise of non-communicable diseases has put further strain on the health sector [53–55]. To improve financial protection and coverage, India is committed to achieving UHC for its population. Three recent important documents, outline this commitment and importantly to establishing a functioning system of HTA to further the UHC agenda: 1) The 12^th^ Five Year Plan (2012 – 2017) issued by the Planning Commission of India [37]; 2) The National Health Policy, issued in 2017; and 3) The NITI Aayog 2017 – 2020 vision document [54,56,57].

Decision-making for resource allocation is often consensus-based nationally and at a state level [44]. Owing to the federal structure of India, and a significantly greater share of public health funding coming from State Governments, states are an essential stakeholder in healthcare decision-making. Some examples of the use of HTA evidence for decision making is seen at the State level (for example, the recent introduction of HPV vaccination in Punjab [77]), however there is no organized system of incorporating such evidence at a broader level [58]. Introduction of the National Health Mission (NHM), under the Ministry of Health and Family Welfare (MoHFW), improved the process of decentralised participatory decision-making. While this process included a detailed situational analysis, the selection of interventions and programs to be subsidised are still heavily guided by its effectiveness, rather than cost effectiveness, or broader impact on social and ethical dimensions [59–61]. The MoHFW has further demonstrated support for evidence based decision-making through the development of: Institutionalised National Health Accounts [62], evidence-based standard treatment guidelines [63], and the establishment of a dedicated HTA body, HTAIn (previously called Medical Technology Assessment Board (MTAB)) [41,48,54]. HTAIn falls under the oversight of the Department of Health Research, within the MoHFW, and will be tasked with developing a robust HTA system to assist decision-makers nationally and at a state level. Additionally, HTAIndia will be responsible for informing the public on HTA findings [44,54,64–68].

#### South Africa’s HTA journey

HTA is yet to be institutionalised or consistently applied in South Africa. The country aims to include this agenda as South Africa moves towards UHC through implementation of the National Health Insurance (NHI). Despite South Africa’s public healthcare system providing services free (or at low cost) at the point of care, low-quality services and insufficient access has led to poor health outcomes and exacerbating inequality. Health expenditure as a percentage of GDP was 8.2% in 2014. This expenditure was split between the private and public sectors at 3.8% and 4.4% respectively despite the private sector servicing only 16–20% of the overall population [69–71]. The inconsistent regulation of the private sector coupled with the increased demand has led to the current oligopoly of private healthcare provider organisations, in particular three large hospital provider groups. In response, a market inquiry into the private health sector was initiated by the Competition Commission of South Africa with provisional findings and recommendations published in July this year. Subject to stakeholder consultation, after which the final report will be released, the provisional recommendations included: ‘changes to the way (medical) scheme options are structured to increase comparability between schemes and increase competition in that market; a system to increase transparency on health outcomes to allow for value purchasing; and a set of interventions to improve competition in the market through a supply side regulator’ [72].

Regulating healthcare costs will be an important factor in the sustainability of NHI, which plans to cover essential services of good quality, for the entire population, free at the point of use [73,74]. Achieving this goal within the resources available, will require a clearly defined health services package utilising tools such as HTA [75]. There have been several policy directives for HTA since 1994. The first policy directive for HTA in South Africa was in 1994 under the ANC National Health Plan. Further policy directives were made in the Framework for Health Technology Policies in 2001, a National Health Technology Strategy in 2009 and the NHI Green Paper in 2011 [76]. However, HTA evidence to inform decision-making is not legislated or consistently utilised. There are some examples in large institutions, such as the revision of the national Essential Medicines List by the National Department of Health and diagnostics by the National Health Laboratory Services, where elements of HTA are applied [73,75,77–81]. Pharmacoeconomic guidelines have been developed by the National Department of Health (NDoH) for external submissions, however this is voluntary and for regulation in the private sector [91]. Critically, the NHI Bill 2018, explicitly links HTA to achieving sustainable UHC providing a stronger policy foundation that was absent in previous attempts to establish HTA. The NHI Bill 2018 states that HTA will inform the health services package delivered under NHI and that a legislated entity, guided by a single national HTA policy, will facilitate coordination of HTA. According to the NHI Bill 2018, ‘The Ministerial Advisory Committee on Health Technology Assessment for National Health Insurance which will be established to advise the Minister on Health Technology Assessment and which will serve as a precursor to the Health Technology Assessment agency that will regularly review the range of health interventions and technology by using the best available evidence on cost -effectiveness, allocative, productive and technical efficiency and Health Technology Assessment’ [74]. No details have been provided about the HTA agency with relation to its position, scope or role in decision-making [73,74]. However, the National Treasury has allocated funding under the NHI programme to support the development of HTA in the 2018 Health Budget [82].

### Cross-case analysis

The results of cross-case analysis are presented under headings aligned with the main categories of the research framework.

#### Category 1 – utilization of HTA in public sector decision-making

The literature revealed that all three countries have formal processes to inform decision-making in terms of either health insurance benefits (China and India) or essential medicine selection (South Africa). No evidence was found to show that the adoption of HTA evidence in decision-making is currently legislated in any of the three countries, however in China HTA has been written into the first draft of Chinese Basic Health Law which is in the process of being reviewed by National People’s Congress and HTA has been highlighted in the National Health Insurance Bill 2018 for South Africa.

#### Category 2 – scope of HTA and availability of guidelines

Based on the available information, both the Indian and South African governments are aiming for HTA to be conducted on a wide range of technologies and interventions. China currently undertake HTA on several different technologies but the full scope is still to be defined. The search provided no evidence of formal guidelines for developing HTA as yet. China and India both have guidelines in development and South Africa will develop appropriate guidelines under the NHI.

#### Category 3 – institutional capacity and human resources supporting HTA

China is the furthest along the HTA journey in terms of the establishment of a national HTA entity and thus have the largest capacity for supporting HTA. Responsibility for HTA falls under the NHFPC of China, guided by the CNHDRC which is also acts the centre of China network for HTA. India have recently established the Health Technology Assessment Board (HTAB, now HTAIn) responsible for the informing the development of an HTA system. South Africa are yet to formulate such a body but aim to develop an HTA entity in the future.

#### Category 4 – governance of the HTA process

The HTA systems in all countries are still in development, as are many of the governance processes. India developed and piloted a conflict of interest policy in 2017. The impact of HTA on policy and decision-making in all countries is still to be determined.

#### Category 5 – requirements for strengthening HTA capacity

China has the most established HTA capacity building initiative. India and South Africa have short courses for HTA available but aim to develop long-term HTA programmes. Several enabling factors were important in the HTA journey of all the three countries, strong stakeholder engagement (all countries), networking at a local level (China), and specific policy links to UHC (India and South Africa).

#### Category 6 – barriers to institutionalising HTA

Shared barriers that were revealed from the information sourced included: Fragmented healthcare and information systems due to multiple insurance schemes (India and China), vertical disease areas (all countries), fiscal federalism (all countries), and large public-private provider split (all countries but especially South Africa), rising healthcare costs (all countries). Lastly all countries are experiencing a rising burden of Non-Communicable Diseases (NCDs), which further complicates priority-setting.

#### Category 7 – future goals for HTA system development

Improving capacity for HTA is a priority area across countries, as is the institutionalisation of HTA and development of HTA methodology. India specifically aims to formulate methodology which takes co-morbidity into account. The routine inclusion of cost-effectiveness evidence in decision-making is a focus for South Africa. All countries aim to continue networking and engagement nationally, regionally and internationally.

## Interpretations

The results of our analyses revealed five critical challenges for strengthening HTA systems in China, India and South Africa: (1) Limited capacity for HTA; (2) Weak data infrastructure; (3) HTA in the context of rising healthcare costs; (4) Coordinating and conducting HTA in fiscal-federal contexts; (5) HTA in context with multiple, large burdens of disease.

Furthermore two conducive factors for strengthening HTA systems were identified: 1) Strong stakeholder engagement; and 2) Establishment of a national coordinating HTA unit

A policy brief and working paper published by Chootipongchaivat *et al*. entitled ‘Conducive factors to the development of Health Technology Assessment in Asia’ identified similar commonalities. The paper provided common problems, conducive factors and recommendations for HTA development in Asia derived from seven country contexts [10].

Each of the critical challenges and two conducive factors identified in our paper will be detailed below with an accompanying recommendation. After which, South-South collaboration for strengthening HTA systems will be explored as this aligns with the inherent nature of this paper and the last recommendation from the working paper above ‘international collaboration’.

### Critical challenges to strengthening HTA systems

#### Limited capacity for HTA

Capacity to perform and utilise HTA is common challenge (see Table 2 below). Although strong institutions for teaching health economics exist in each of the three countries, further specialised training in HTA and priority-setting is needed in all instances. Consequently, all countries aim to increase short-term and long-term capacity. **Recommendation**: A framework for approaching capacity building in LMICs for evidence-based priority setting was developed by Li *et al*. (2017). Capacity building should encompass a variety of methods and approaches, adapted for different audiences and country settings [83].

#### Weak data infrastructure

Robust HTA mechanisms are facilitated by strong information systems and so improving data infrastructure is critical, although this may have to be developed in parallel to building an institutionalised HTA framework. **Recommendation**: Decisions are inevitable despite the level of evidence that currently exists, however a commitment to using HTA in policymaking may also drive improvements in data collection, which may have wider benefits. Developing tools, such as a costing database that could be shared nationally, or country-specific values for quality of life, could improve consistency and the quality of analyses.

#### HTA in the context of rising healthcare costs

High OOPs (in China and India), poor-quality services, rising prices in the private sector, and high pharmaceutical prices in all countries have contributed to a large private-public provider split. Private expenditure on health as a percentage of total health expenditure in 2014 for China, India and South Africa was 44%, 70% and 52% respectively [53]. Furthermore high levels of inequality are experienced [84]. **Recommendation**: Developing a fair, yet affordable, benefit package is this context is especially difficult [85]. HTA evidence could aid in price negotiation and be utilised to inform the best allocation of resources, promoting the provision of equitable healthcare services [86].

#### Coordinating and conducting HTA in fiscal-federal contexts

Healthcare service delivery, as well as resource allocation, is devolved to various levels of government in China, India and South Africa. Multiple stakeholders and budget holders can add to the complexity of priority-setting in fiscal-federal contexts. This highlights the importance of federal entities engaging with national policymakers (and vice-versa). **Recommendation**: Establishing a national entity to coordinate HTA and priority setting, in the context of fiscal federalism, could aid with better price negotiation, improve inequities, consolidate capacity and reduce duplication of work.

#### HTA in contexts with multiple, large burdens of disease

An epidemiological transition to Non-Communicable Diseases has been experienced across many LMICs [87]. This coupled with a remaining burden of communicable diseases, has placed great strain on the health systems. Subsequently, many patients suffer from multiple conditions, which can complicate clinical pathways and treatment. **Recommendation**: HTAs are traditionally performed for single conditions or technologies however, methodology to address the complexities associated with co-morbidities need to be developed.

### Conducive factors for strengthening HTA systems

#### Strong stakeholder engagement

Country experiences in China and India revealed the importance of high-level buy in successful implementation of HTA processes – engagement and capacity building with policymakers is fundamental. This is enabled by building credibility of units or organisations producing and/or coordinating HTA. Engagement with the stakeholders such as the public, private sector and societies will be an important aspect for all countries, to provide valuable input into the decision-making process and facilitate the acceptability and implementation of decisions. **Recommendation**: Development of mechanisms and process to facilitate systematic stakeholder engagement.

#### Establishment of a national coordinating HTA unit

China, India and South Africa have committed to developing strong HTA systems and each country has demonstrated this commitment through recently published policies. Each country has either recently established a dedicated HTA group or unit (HTAIn in India), formed a network hub to strengthen HTA practice (China National Health Development Research Center – CNHDRC) or provided for the establishment of an advisory group on HTA (Ministerial Advisory Committee on Health Technology Assessment for National Health Insurance in South Africa).

**Recommendation**: The scope and structure of a national HTA unit or agency is important during the development of HTA systems and is context specific. Relevant questions include: should the focal unit be closely aligned with government health departments as in China or India, or a more independent entity? To what extent should the agency be involved in decision-making – directly (as NICE in the UK), or through the provision recommendations to subsequently acted on by relevant government departments and ministers? Finally, what are the benefits and downsides of different organisational approaches for developing the core assessments needed as part of the HTA process, which can be done ‘in-house’ (i.e. within the identified focal unit); entirely outsourced to external evaluation groups, such as academic institutions; or through a mixed model?

## South-South collaboration and collaborative research to strengthen knowledge and HTA utilisation for better healthcare decision-making

Although the analysis of the available published literature resulted in important insights, the workshop materials and the collaborative process involved in producing this paper provided additional information, from first-hand sources on common challenges and lessons. The presence of government representatives, as well as national and international experts at the workshop, facilitated rich discussions resulting in beneficial shared knowledge, which was further enhanced during the collaboration of the paper. Country collaborations have grown over the decades, evolving from one-way donor dependency to promotion of self-reliance nationally and amongst countries. Additionally, countries recognised the benefits of learning and sharing experiences across countries with more comparable contexts [88]. Triangular Collaboration (North-South-South) is another beneficial mechanism whereby one high-income country supports collaboration and capacity building between countries of the Global South often through financial or technical contributions [89]. For this type of collaboration, it is important that agendas and development plans primarily remain under the remit of the South-South partners, to prevent the engagement becoming a pseudo North-South relationship [90]. This approach is fundamental in the work of networks such as iDSI where knowledge sharing and technical support are demand driven and partnerships aim to provide relevant and pragmatic assistance [13].

Continued knowledge sharing and capacity building through south-south collaboration and collaborative research on particular topics, for example strengthening HTA systems in the context of fiscal federalism or multi-morbidities, could assist Global South countries in navigating these common challenges. Similarly south-south collaboration could support countries to leverage enabling factors for HTA strengthening. Further knowledge sharing could be facilitated through collaborations such as: staff visits and exchanges between countries; the development of an online platform to share all types of materials such as ‘How-to guides’ or clinical guidelines; workshops and meetings facilitating knowledge sharing; and further collaborative research on key topic areas. These practices could be mutually beneficial for all, especially aiding countries with data and capacity limitations and enabling evidence-based decision making on a wider range of topics than would be possible in isolation.

### Limitations

The authors acknowledge the vast variation between countries within the Global South and that China, India and South Africa may not reflect typical characteristics of these countries, limiting the potential generalisability of the findings. Case selection was predominantly determined by the accessibility to nuanced information and country experts to illicit pragmatic lessons as well as collaborative approach. Networking with the other two BRICS countries and other countries in the Global South could help facilitate similar collaborations to explore similar challenges and enablers to strengthening HTA systems in those contexts.

## Conclusion

The healthcare systems in each country are fragmented, exacerbating existing inequalities. However, all countries are on the path to UHC and aim to institutionalise HTA, build capacity and develop HTA methodology. China, India and South Africa have all committed to strengthening their priority setting and resource allocation institutions. They are all at different stages in the process of establishing more formalised HTA systems. The countries share many similar challenges such as: 1) Minimal expertise to conduct robust HTA analyses; 2) Weak data infrastructure; 3) Fragmented healthcare systems; 4) Fiscal-federal contexts; and 5) Massive growth in non-communicable diseases, a strong driver of higher healthcare costs. Two conducive factors to strengthening HTA systems were identified; strong engagement with policy and decision makers, and the establishment of a national HTA entity. There are several actions that can be taken to address the above challenges and leverage conducive factors including, comprehensive capacity building initiatives and developing a well-defined scope for HTA entities. Collaboration (with national and international experts) through a supportive network can aid in the progress towards the institutionalisation of HTA. Collaborative research and engagement, especially between countries of the Global South, can provide a beneficial platform to share knowledge, which is applicable and pragmatic.

## Supplementary Material

Supplemental Material

## Paper context

Health Technology Assessment (HTA) and explicit priority setting has gained increased acknowledgement from policymakers in countries of the Global South. Especially as countries, such as China, India and South Africa move towards UHC to address inequities and provide a sustainable benefits package. The countries experience similar challenges, collaboration across countries could elicit shareable insights for strengthening HTA systems and capacity that are useful and pragmatic.

## Appendix A. Search strategy for systematic search component

**Identification of documents**

The following search terms were applied to the title and/or abstract for each country (China, Brazil and South Africa):

1. ‘health technology assessment’ OR ‘HTA’ OR ‘priority setting’ OR ‘priority-setting’
2. ‘evidence-based decision making’ OR ‘evidence based decision-making’ OR ‘evidence based decision making’
3. ‘essential medicines list’ OR ‘essential drugs list’ OR ‘EML’ OR ‘EDL’
4. ‘universal health coverage’ OR ‘UHC’
5. ‘health’ AND ‘benefit’ AND ‘package’
6. (‘universal health coverage’ OR ‘UHC’) AND ‘health’ AND ‘benefit’ AND ‘package’

The database search provided for the identification of 285, 348 and 285 records for China, India and South Africa respectively. A number of records (China = 17; India = 13; South Africa = 7), were identified through a grey literature iterative search on advice from country representatives.

**Screening of documents**

The records for each country were screened and duplicates were removed (China = 90; India = 88; South Africa = 51). Updated search (China = 7; India = 27; South Africa = 12). Records were further screened using the following inclusion and exclusion criteria (see Table A1 below):

**Table A1.** Inclusion and exclusion criteria utilised for screening articles.

Inclusion Criteria	Exclusion Criteria
Full text article access	Abstract only available
Articles on China, India or South Africa	Articles on any other country
Articles on healthcare	Articles on another field other than healthcare
	Articles on: Drug prescribing patterns; Medical education not directly related to priority-setting, HTA or evidence-based decision making for heath; A particular disease area or intervention
	Clinical trials
	Systematic reviews
	Cost effectiveness studies or HTAs

Two hundred and twelve records were screened for China and 51 records excluded. The screening of the 272 records for India resulted in 111 records being excluded. Finally, 239 records for South Africa were screened and 122 records excluded.

**Full-Text Review for Eligibility**

For each country, the remaining records (China = 161; India = 162; and South Africa = 117) underwent a full text review to assess eligibility for inclusion in the synthesis.

**Table:**

Inclusion Criteria	Exclusion Criteria
Topic of the article relates to the practice of the following in the public health care system:(1) Priority setting(2) Evidence-based decision making;(3) Health Technology Assessment	Articles about: Priority setting for research Methodology only; Healthcare financing only; Financial protection only; Healthcare access or utilization only.

Review of the full text of documents resulted in the exclusion of 121 records each for China and India and 92 records for South Africa. China – 34 records excluded, leaving only 5 included.

**Documents Included for Analysis**

After the screening and full text assessment processes, 40 documents were included for China, 41 documents for India and 25 documents for South Africa (total 106). Four articles were relevant to multiple countries and thus duplicated across two or three of the countries (six duplicates in total). The removal of these duplicates resulted in 100 documents included for analysis. Original search was run in 2017 thus an updated search was conducted in August 2018, resulting in a further inclusion of 5, 4 and 6 documents for China, India, ad South Africa respectively.

**Appraisal of included documents**

All documents were included and did not undergo further appraisal as data extraction was guided directly by the extraction framework. However where there were multiple sources of information per framework sub-category, the most up to date information from the best-quality source was utilised i.e. Journal article, book chapter, government publication over media articles, or presentations.
