# Supplementary material for: Strengthening health technology assessment systems in the global south: a comparative analysis of the HTA journeys of China, India and South Africa
Source: Glob Health Action. 2018 Oct 17;11(1):1527556. doi: 10.1080/16549716.2018.1527556 (PMC6197020; doi:10.1080/16549716.2018.1527556)
Supplement: Supplemental Material [file ZGHA_A_1527556_SM6628.zip › S1_Search Strategy_clean version_10092018.pdf]

S1 – Search strategy for systematic search component

Identification of documents

The following search terms were applied to the title and/or abstract for each country (China, Brazil and South Africa):

1. "health technology assessment" OR "HTA" OR "priority setting" OR "priority-setting"
2. "evidence-based decision making" OR "evidence based decision-making" OR "evidence based decision making"
3. "essential medicines list" OR "essential drugs list" OR "EML" OR "EDL"
4. "universal health coverage" OR "UHC"
5. "health" AND "benefit" AND "package"
6. ("universal health coverage" OR "UHC") AND "health" AND "benefit" AND "package"

The database search provided for the identification of 285, 348 and 285 records for China, India and South Africa respectively. A number of records (China = 17; India = 13; South Africa = 7), were identified through a grey literature iterative search on advice from country representatives.

Screening of documents

The records for each country were screened and duplicates were removed (China = 90; India = 88; South Africa = 51). Updated search (China = 7; India = 27; South Africa = 12). Records were further screened using the following inclusion and exclusion criteria:

Table 1: Inclusion and exclusion criteria utilised for screening articles

**Inclusion Criteria**	**Exclusion Criteria**
Full text article access	Abstract only available
Articles on China, India or South Africa	Articles on any other country
Articles on healthcare	Articles on another field other than healthcare
	Articles on:<br>➢ Drug prescribing patterns;<br>➢ Medical education not directly related to priority-setting, HTA or evidence-based decision making for heath;<br>➢ A particular disease area or intervention
	Clinical trials
	Systematic reviews
	Cost effectiveness studies or HTAs

Two hundred and twelve records were screened for China and 51 records excluded. The screening of the 272 records for India resulted in 111 records being excluded. Finally, 239 records for South Africa were screened and 122 records excluded.

Full-Text Review for Eligibility

For each country, the remaining records (China = 161; India = 162; and South Africa = 117) underwent a full text review to assess eligibility for inclusion in the synthesis.

Inclusion Criteria	Exclusion Criteria
Topic of the article relates to the practice of the following in the public health care system:<br>➢ Priority setting<br>➢ Evidence-based decision making;<br>➢ Health Technology Assessment	Articles about:<br>➢ Priority setting for research<br>➢ Methodology only;<br>➢ Healthcare financing only;<br>➢ Financial protection only;<br>➢ Healthcare access or utilization only.

Review of the full text of documents resulted in the exclusion of 121 records each for China and India and 92 records for South Africa. China – 34 records excluded, leaving only 5 included.

Documents Included for Analysis

After the screening and full text assessment processes, 40 documents were included for China, 41 documents for India and 25 documents for South Africa (total 106). Four articles were relevant to multiple countries and thus duplicated across two or three of the countries (six duplicates in total). The removal of these duplicates resulted in 100 documents included for analysis. Original search was run in 2017 with updated search conducted in August 2018, resulting in a further inclusion of 5, 4 and 6 documents for China, India, ad South Africa respectively.

Appraisal of included documents

All documents were included and did not undergo further appraisal as data extraction was guided directly by the extraction framework. However where there were multiple sources of information per framework sub-category, the most up to date information from the best quality source was utilised i.e. Journal article, book chapter, government publication over media articles, or presentations.
