# Supplementary material for: Strengthening health technology assessment systems in the global south: a comparative analysis of the HTA journeys of China, India and South Africa
Source: Glob Health Action. 2018 Oct 17;11(1):1527556. doi: 10.1080/16549716.2018.1527556 (PMC6197020; doi:10.1080/16549716.2018.1527556)
Supplement: Supplemental Material [file ZGHA_A_1527556_SM6628.zip › S2 - Data extraction_clean version_10092018.pdf]

S2: Completed framework after data extraction

Health Technology Assessment in China, India and South Africa

Supplementary Material for:

CAPACITY BUILDING ARTICLE in *Global Health Action*

Strengthening Health Technology Assessment Systems in the Global South: A Comparative Analysis of the HTA journeys of China, India and South Africa.

Kim MacQuilkan[1], Peter Baker[2], Laura Downey[2], Francis Ruiz[2], Kalipso Chalkidou[2], Shankar Prinja[3], Kun Zhao[4], Thomas Wilkinson[1], Amanda Glassman[5], Karen Hofman[1]

[1] Priority Cost Effective Lessons for System Strengthening South Africa (PRICELESS SA), School of Public Health, University of Witwatersrand, Faculty of Health Sciences, Johannesburg, South Africa.

[2]Global Health and Development Group, Institute of Global Health Innovation, Imperial College London, London, United Kingdom.

[3]School of Public Health, Post Graduate Institute of Medical Education and Research (PGIMER), Chandigarh, India

[4]Division of Health Technology Assessment and Policy Evaluation, China National Health Development Research Center (CHNHDR), Ministry of Health, Beijing, China

[5]Center for Global Development, Washington DC, United States of America

Corresponding Author: Kim MacQuilkan

Priority Cost Effective Lessons for System Strengthening South Africa (PRICELESS SA),

Room 231, School of Public Health, Faculty of Health Sciences, University of Witwatersrand.

27 St. Andrews Road, Parktown, Johannesburg, South Africa, 2193

S2: Completed framework after data extraction – Health Technology Assessment in China, India and South Africa

Category 1 - Utilization of HTA in public sector decision-making

Sub-Category	CHINA	INDIA	SOUTH AFRICA
1A) Formal 'information-gathering process' for decision making	• Different health insurance schemes have different processes for determining benefits [1–8].<br>• One of the units conducting HTA on behalf of the Chinese government is the China National Health Development Research Center (CNHDRC), under the responsibility of the National Health and Family Planning Commission (NHFPC) [9–12] .	• Different health insurance schemes have different processes for determining benefits, as do different government vertical programs [13–15].<br>• Ministerial appointed National Essential Medicines List Committee (NEMLC), responsible for amending Essential Medicines List (EML) [16,17]. Higher level expert groups (HLEG) also convene to advise on inclusions and exclusions, within various program packages [18].<br>• National Technical Advisory group on Immunization have a formal process for generation and synthesis of evidence to inform national vaccine uptake [19].	• Formal selection process for pharmaceuticals only. Ministerial appointed National Essential Medicines List Committee (NEMLC), responsible for amending Essential Medicines List (EML) and Standard Treatment Guidelines (STGs) [20,21]. On a provincial, district and local level – non-statuary Pharmaceutical and Therapeutics Committees (PTCs) responsible for formulating relevant formularies[22,23].<br>• Decisions on medicine selection at all levels not formally informed by HTA but primarily based on safety, clinical effectiveness, availability, budget impact with cost-effectiveness less consistently utilised [20,21].<br>• National Health Laboratory Services (NHLS) have an HTA unit to conduct HTA for some laboratory services and diagnostic technologies [24].
1B) Legislative requirements for considering HTA findings	• HTA has been written into the first draft of Chinese Basic Health Law which is in the process of being reviewed by National People's Congress. China Health Policy and Technology Assessment network (China HTA network), with CNHDRC as coordinator has been established to aid decision-makers and potentially inform policy [10,11,25–27].	• No formal legislative requirement to consider HTA findings [28]. However, the necessity for HTA to inform decision-making has been recognised in the 12th 5-year plan [18], the National Health Policy [29] and the Niti Aayog 2017 – 2020 vision document [30].	• Not currently - The National Health Insurance (NHI) White Paper released 2015 updated in 2017 and the Bill 2018 states the need for HTA to inform public funding decision-making related to all technologies and the proposal of an independent entity to coordinate this process [31,32]. The National Treasury allocated funding for HTA under NHI in the 2018 Vote 16 Health Budget [33].

Category 1 - Utilization of HTA in public sector decision-making (Cont.)

Sub-Category	CHINA	INDIA	SOUTH AFRICA
1C) Purposes of undertaking HTA	● Regulation of medical practices<br>● Pricing negotiation for high cost drugs<br>● Benefit package decisions for insurance schemes [8,11,25,26,34]	● HTA will be used to inform resource allocation and reimbursement decisions [35,36]	● **Current** – Some HTA utilised at the NHLS to inform pricing and reimbursement decisions for selected diagnostics [24], some financial information considered in medicine selection to the national EML within the Essential Drugs Programme (EDP) [37].<br>● **Future** – To inform decision making for public funding of all types of interventions, services and technologies under the NHI [31,32].
1D) Types of technologies or interventions assessed	● Pharmaceuticals, devices, vaccines, interventions [8,11,34,38]	● HTA will be utilised to assess drugs, devices, and health programs [35,36]	● **Current** –Diagnostic technologies and laboratory services (NHLS) [24], medicine EDP [37].<br>● **Future**: NHI White Paper 2017: "HTA will inform prioritisation, selection, distribution, management and introduction of interventions for health promotion, disease prevention, diagnosis, treatment and rehabilitation" [31,32]

Category 2 - Scope of HTA and availability of guidelines

Sub-Category	CHINA	INDIA	SOUTH AFRICA
2A) Aspects considered in HTA	In development by China National Health Development Research Center (CNHDRC) [11]	HTA process will consider three broad areas: clinical and cost-effectiveness, reduction in OOP expenditure, and impact on equity in utilization of services [28,35,36].	Specific aspects that will be considered in the HTA process under NHI have not be explicitly stated in the NHI White Paper 2017 or NHI Bill 2018 [31,32].
2B) Guidelines for developing HTA	In development by China HTA network, National HTA Toolkit [11,38].	A reference case for economic evaluation and methods manual for the conduct of HTA is in development [35,36].	Ministerial Advisory Committee on Health Technology Assessment for National Health Insurance will advise on the development of an HTA agency and HTA system [31,32].

Category 3 - Institutional capacity and human resources supporting HTA

Sub-Category	CHINA	INDIA	SOUTH AFRICA
**3A) National HTA organization**	China National Health Development Research Center (CNHDRC) under the National Health and Family Planning Commission (NHFPC). Responsibility for HTA lies with the NHFPC [9–11,27,38]. The HTA division of CNHDRC will soon be designated the focal HTA organisation of China [11,12].	The Department Health Research (DHR) has been given mandate to establish a functioning system of HTA in India, as per 12th 5 year plan [18]. DHR established the Medical Technology Assessment Board (MTAB), a dedicated HTA board tasked with establishing a robust HTA system [28,29,35,36]. Following this, a dedicated entity HTAIn (Health Technology Assessment in India) was formed in early 2018 [39].	● Currently no single national HTA organization [31,32,40].<br>● Future, under NHI, establishment of a legislated HTA entity [31,32].
**3B) Number of staff members in HTA organizations**	HTA research units part of HTA Hub with number of staff [11]:<br>● CNHDRC HTA unit: **12-18**<br>● Health Technology Assessment Key Lab at Fudan University: **3-5**<br>● Health Technology Assessment Center at the Institute of Health Service and Medical Information, Shanghai: **5-8**<br>● Health Technology Assessment center at Peking University: **5-9**	At present, core team within the MTAB/HTAIn secretariat employs dedicated staff, covering both technical and administrative roles. A network of 15 institutions has been identified to engage in HTA-related activities, and **3-4** staff from each of these institutions will undertake intensive training [35,41].	National HTA entity still to be established under NHI [31,32].
**3C) Requests for HTAs**	HTA research units receive from local, provincial and national decision-makers. In the Hub framework, requests will be directed to the steering committee [11,12,42].	A number of requests have been received by the DHR to undertake HTA from union and state ministry departments. A total of 8 topics have been prioritised in the first instance [41,43] .	Not yet specified.
**3D) Professionals involved in HTA preparation and decision making**	Specialists in Economics, Medicine, Sociology, HTA and Epidemiology [11].	The Technical Appraisal committee for HTA is a multidisciplinary body with expert economists, clinicians, researchers, social scientists, and policy experts [36,41]	The future Ministerial Advisory Committee on Health Technology Assessment for National Health Insurance will be multidisciplinary team. NHI White Paper 2017 states the Committee will comprise medical and other experts [31].

Category 4 - Governance of the HTA process

Sub-Category	CHINA	INDIA	SOUTH AFRICA
4A) Conflict of interest declaration	To be developed through China HTA network coordinated by CNHDRC [11,12].	A formal conflict of interest declaration has been developed and trialled in the first MTAB stakeholder meeting, held in July 2017 [36,43].	To be developed by Ministerial Advisory Committee on Health Technology Assessment for National Health Insurance and/or HTA entity [31,32].
4B) Communicating the outcomes of HTA	Mechanisms for disseminating findings to be developed through China HTA network coordinated by CNHDRC [11,12] .	The first HTA still to be completed thus mechanisms for communicating outcomes of HTA in development [28,36,41].	HTA agency will provide evidence/recommendations to the NHI Benefits Advisory Committee. Specifics still to be developed under the advice of the Ministerial Advisory Committee [31,32].
4C) HTA entity involvement in policy and decision making	Advisory - To be developed through China HTA network coordinated by CNHDRC [11,12].	At this stage, no HTA has been undertaken to completion and hence it is not possible to comment on the connection between HTA and decision making [41]	HTA agency will provide evidence/recommendations to the NHI Benefits Advisory Committee [31,32].
**4D) Stakeholder engagement – civil society, public in the HTA process**	Mechanisms and processes for stakeholder engagement to developed through China HTA network coordinated by CNHDRC [11,12].	Stakeholder meetings have been held for all HTA analyses being undertaken. Stakeholders are required to register formally via a link on the DHR website [43,44] .	Not specified in NHI White Paper 2017 or NHI Bill 2018 [31,32] .

Category 5 - Requirements for strengthening HTA capacity

5A) Main barriers for producing HTA and using HTA findings in decision making	• Decentralisation, multiple insurance schemes, lack of relevant legislation [8,45].	A number of barriers to this effort exist. The most challenging of these include a lack of human resources and skills to undertake HTA and a lack of quality data to input into analyses [28,35,36].	• There is no single national policy for HTA or dedicated legislated entity.<br>• Human resources and capacity: Medicine selection is the responsibility of the National Essential Medicines Committee [NEMLC] of which the Essential Drug Programme (EDP) is the secretariat. Analyses to inform decision-making are conducted by four sub-committees comprising external experts. However, due the large volume of reviews required and the reliance on external experts, analyses are primarily focused on clinical effectiveness [20]. Limited HTA capacity in the country [46,47].

Category 5 Continued- Requirements for strengthening HTA capacity

Sub-Category	CHINA	INDIA	SOUTH AFRICA
5B) Enablers in the progress towards institutionalisation of HTA	• Strong engagement with policymakers [11]<br>• Existence of units with expertise in HTA further enhanced by the establishment of the HTA China Network coordinated by CNHDRC [10,11,27,38]	• Strong engagement with policymakers [36]<br>• Support from local and international experts [[36,48]	• Strong engagement with policymakers [46]<br>• Strong policy foundation for HTA under the NHI [31,32]<br>• Support from local and international stakeholders [46]
5B) Academic or training programmes to support capacity building for HTA	• Various universities and research institutions as well as the China HTA Network [11,12,38].<br>• China National Health Development Research Center (CNHDRC is supporting the strengthening of HTA processes [11,27,38].	• Intensive training courses for HTAIndia technical partners on the conduct of Health Technology Assessment have been run in Kerala and Chandigarh to date. Such course will continue to be run in order to incrementally build upon skills and knowledge to undertake HTA[49].<br>• Two short online courses have also been developed by the School of Public Health at PGIMER Chandigarh to allow students to access content remotely and complete certification in basic economics and advanced economics for HTA [50].	• There are currently a few post-graduate degree programmes in South Africa focusing on health economics, although none specifically on HTA. However there is a 3-month, postgraduate certificate course available from University of Stellenbosch explicitly on HTA for medical technologies [51]] .<br>• MPH programmes in health economics are offered at the University of Cape Town [52] and as of 2018, a MPH in health economics at the University of Witwatersrand which comprises several specialised modules covering aspects related to HTA [53] .

Category 6 - Barriers to Institutionalising HTA

Sub-Category	CHINA	INDIA	SOUTH AFRICA
**Barriers to institutionalising HTA**	• Weak awareness in HTA among policy makers at local levels<br>• Multiple insurance schemes with different pools and payers [8,11,45,46];<br>• Fragmented data also due to different insurance schemes [8,11,45];<br>• High pharmaceutical prices [8,34,54];<br>• Fiscal federalism – different priorities at various levels of government [11,45,46].	• High OOP and private sector expenditure. How to develop a basic benefit package that prevents catastrophic expenditure but is affordable [55–57]<br>• Low government expenditure on healthcare [45,56].<br>• How to address growing burden of NCDs and conduct HTA in a context of multi-morbidities [46];<br>• Fiscal federalism – different priorities at various levels of government [36,46].	• Lack of capacity to conduct HTA [31,32,46];<br>• Fiscal Federal context – need buy-in at provincial and district levels and common alignment of budgets for health technologies [46];<br>• Vertical programmes – need for cohesion across programmes and divisions in terms of policy and clinical guidelines [46];<br>• Strong private sector leading to rising costs [58,p.27,59] .

Category 7 - Future Goals for HTA system development

Sub-Category	CHINA	INDIA	SOUTH AFRICA
**Future goals for strengthening**	1. Helping develop institutional structure and mechanism of HTA;<br>2. Conducting research for the HTA handbook including standardised processes set at the national level;<br>3. Conducting research for the value judgement for pricing negotiation at the national level;<br>4. Continually running the national HTA network under the leadership of NHFPC;<br>5. Continue to undertake long-term capacity building mentor programmes, which require universities to be involved.<br>[10,11,27,38,42].	1. Need to build up capacity for HTA;<br>2. Identifying data gaps;<br>3. Creating relevant database and supporting information systems to facilitate HTA;<br>4. Converting data into useful information for decision- and policymakers;<br>5. Strategies to consider multi-morbidities;<br>6. Development of an Indian Reference Case;<br>7. Continued synergy and engagement with policymakers<br>[28,36,46].	1. Establishing the Ministerial Advisory Committee on Health Technology Assessment for National Health Insurance and subsequent HTA entity;<br>2. More focus on inclusion of cost-effectiveness evidence into decision-making;<br>3. Facilitate the use HTA to inform the health services package under NHI;<br>4. Build capacity for conducting HTA through the establishment for degrees and courses;<br>5. Conclusion of the market inquiry into the private healthcare sector to evaluate rising healthcare costs.<br>[31,32,40,58,p.27,59].

REFERENCES

[1] Gross A. Reimbursement in China [Internet]. 2010 [cited 2017 Aug 24]. Available from: www.raps.org/WorkArea/DownloadAsset.aspx?id=3268.

[2] Mossialos E, Ge Y, Hu J, et al. Pharmaceutical Policy in China: Challenges and Opportunities for Reform. [Internet]. Copenhagen: World Health Organization; 2016. Available from: http://www.euro.who.int/__data/assets/pdf_file/0020/320465/Pharmaceutical-policy-China-challenges-opportunities-reform.pdf.

[3] Barber S, Yao L. Health Insurance Systems in China: A Briefing Note. [Internet]. Geneva: World Health Organization; 2010. Report No.: Background Paper 37. Available from: http://www.who.int/healthsystems/topics/financing/healthreport/37ChinaB_YFINAL.pdf.

[4] Yu H. Universal health insurance coverage for 1.3 billion people: What accounts for China's success? Health Policy. 2015;119:1145–1152.

[5] Yang W, Wu X. Providing Comprehensive Health Insurance Coverage in Rural China: a Critical Appraisal of the New Cooperative Medical Scheme and Ways Forward. Global Policy. 2017;8:110–116.

[6] Yuan B, Jian W, He L, et al. The role of health system governance in strengthening the rural health insurance system in China. International Journal for Equity in Health [Internet]. 2017 [cited 2018 Sep 4];16. Available from: http://equityhealthj.biomedcentral.com/articles/10.1186/s12939-017-0542-x.

[7] Barber SL, Huang B, Santoso B, et al. The reform of the essential medicines system in China: a comprehensive approach to universal coverage. Journal of Global Health [Internet]. 2013 [cited 2018 Sep 4];3. Available from: http://www.jogh.org/documents/issue201301/V3_Barber.pdf.

[8] Kennedy-Martin T, Mitchell BD, Boye KS, et al. The Health Technology Assessment Environment in Mainland China, Japan, South Korea, and Taiwan—Implications for the Evaluation of Diabetes Mellitus Therapies. Value in Health Regional Issues. 2014;3:108–116.

[9] Qing X. Towards the "International Examination Room" China Health Technology Assessment submitted a high score [Internet]. 2017 [cited 2018 Feb 10]. Available from: http://www.cn-healthcare.com/article/20171110/content-497125.html.

[10] Chalkidou K. Launch of China Health Policy and Technology Assessment Network under the auspices of NHFPC [Internet]. 2017 [cited 2017 Oct 21]. Available from: http://www.idsihealth.org/blog/launch-of-china-health-policy-and-technology-assessment-network-under-auspices-of-national-health-and-family-planning-committee/.

[11] Zhao K. China HTA Development [Internet]. Johannesburg; 2016. Available from: http://www.idsihealth.org/blog/idsi-south-south-knowledge-sharing-workshops-johannesburg-south-africa/.

[12] Li M. China HTA Hub [Internet]. Johannesburg, South Africa; 2016. Available from: http://www.idsihealth.org/blog/idsi-south-south-knowledge-sharing-workshops-johannesburg-south-africa/.

[13] MoHFW (Ministry of Health and Family Welfare). National Health Insurance Schemes 2016. [Internet]. 2016 [cited 2017 Aug 31]. Available from: https://www.nhp.gov.in/national-health-insurance-schemes_pg#Universal Health Insurance Scheme (UHIS).

[14] Prinja S, Chauhan AS, Karan A, et al. Impact of Publicly Financed Health Insurance Schemes on Healthcare Utilization and Financial Risk Protection in India: A Systematic Review. Xia C-Y, editor. PLOS ONE. 2017;12:e0170996.

[15] Central Government Health Scheme (CGHS). About CGHS [Internet]. 2017 [cited 2017 Aug 31]. Available from: http://msotransparent.nic.in/cghsnew/index.asp.

[16] World Health Organization. National List of Essential Medicines 2015 India [Internet]. [cited 2017 Sep 22]. Available from: http://apps.who.int/medicinedocs/documents/s23088en/s23088en.pdf.

[17] Report of the Core-Committee for Revision of National List of Essential Medicines (NLEM) [Internet]. World Health Organization; [cited 2017 Sep 22]. Available from: http://apps.who.int/medicinedocs/documents/s23087en/s23087en.pdf.

[18] Government of India, Planning Commission. Twelfth Five Year Plan (2012 - 2017) - Social Sectors (Volume III) [Internet]. Government of India; 2013. Available from: http://planningcommission.gov.in/plans/planrel/12thplan/pdf/12fyp_vol2.pdf.

[19] John TJ. India's National Technical Advisory Group on Immunisation. Vaccine. 2010;28:A88–A90.

[20] Perumal-Pillay VA, Suleman F. Selection of essential medicines for South Africa - An analysis of in-depth interviews with national essential medicines list committee members. BMC Health Services Research [Internet]. 2017;17. Available from:

https://www.scopus.com/inward/record.uri?eid=2-s2.0-85008441772&doi=10.1186%2fs12913-016-1946-9&partnerID=40&md5=c5274346f816f54947da4937203f2de0.

[21] Perumal-Pillay VA, Suleman F. Quantitative evaluation of essential medicines lists: the South African case study. BMC Health Services Research [Internet]. 2016 [cited 2018 Sep 4];16. Available from: http://bmchealthservres.biomedcentral.com/articles/10.1186/s12913-016-1937-x.

[22] Matlala M, Gous AGS, Godman B, et al. Structure and activities of pharmacy and therapeutics committees among public hospitals in South Africa; findings and implications. Expert Review of Clinical Pharmacology. 2017;10:1273–1280.

[23] National Department of Health. The National Policy for the Establishment and Functioning of Pharmaceutical and Therapeutics Committees in South Africa. [Internet]. 2015 [cited 2017 Aug 31]. Available from: http://www.health.gov.za/index.php/pharmaceutical-and-therapeutics-committees?download=1462:policy-pharmaceutical-and-therapeutics-committees-2015.

[24] National Health Laboratory Services. Health Technology Assessment Unit [Internet]. 2017 [cited 2017 Nov 10]. Available from: http://www.nhls.ac.za/?page=hta_unit&id=87.

[25] Zhou W, Zheng Y, Morton A, et al. Delivering universal health coverage for an aging population: An analysis of the Chinese rural health insurance program [Internet]. 2018. Available from: https://www.scopus.com/inward/record.uri?eid=2-s2.0-85050702213&doi=10.1016%2fj.chieco.2018.07.007&partnerID=40&md5=c76376f5a9361f9cf07f51cb9ea1b984.

[26] Health Technology Assessment and Its Applications in China [Internet]. International Society for Pharmacoeconomics and Outcomes Research (ISPOR); 2017 [cited 2018 Oct 2]. Available from: http://press.ispor.org/asia/index.php/2017/10/19/health-technology-assessment-and-its-applications-in-china/.

[27] Ruiz F, Krajenbrink E. Launch of new Chinese health ministry should help develop UK-China partnership in Health Technology Assessment [Internet]. Available from: https://www.idsihealth.org/blog/launch-of-new-chinese-health-ministry-should-help-develop-uk-china-partnership-in-health-technology-assessment.

[28] Prinja S, Downey LE, Gauba VK, et al. Health Technology Assessment for Policy Making in India: Current Scenario and Way Forward. PharmacoEconomics - Open. 2018;2:1–3.

[29] Ministry of Health and Family Welfare. National Health Policy 2017 [Internet]. 2017 [cited 2017 Oct 13]. Available from: Available from https://www.nhp.gov.in//NHPfiles/national_health_policy_2017.pdf.

[30] NITI Aayog. Three year action agenda, 2017-18 to 2019-20 [Internet]. 2017 [cited 2017 Nov 15]. Available from: http://niti.gov.in/content/three-year-action-agenda-2017-18-2019-20.

[31] National Department of Health. National Health Insurance for South Africa: Towards Universal Health Coverage White Paper 2017 [Internet]. 2017 [cited 2017 Oct 10]. Available from: http://www.health.gov.za/index.php/component/phocadownload/category/383.

[32] National Department of Health. National Health Insurance Bill 2018 [Internet]. 2018 [cited 2018 Aug 24]. Available from: file:///C:/Users/kemac/Downloads/national%20health%20insurance%20bill%202018.pdf.

[33] National Treasury of South Africa. 2018 Budget - Estimates of Expenditure: Vite 16 Health [Internet]. 2018 [cited 2018 Aug 24]. Available from: http://www.treasury.gov.za/documents/national%20budget/2018/enebooklets/Vote%2016%20Health.pdf.

[34] Hu J, Mossialos E. Pharmaceutical pricing and reimbursement in China: When the whole is less than the sum of its parts. Health Policy. 2016;120:519–534.

[35] Downey LE, Mehndiratta A, Grover A, et al. Institutionalising health technology assessment: establishing the Medical Technology Assessment Board in India. BMJ Global Health. 2017;2:e000259.

[36] Downey LE. MTAB and HTA in India [Internet]. Johannesburg; 2016. Available from: Available from: http://www.idsihealth.org/blog/idsi-south-south-knowledge-sharing-workshops-johannesburg-south-africa/.

[37] National Department of Health. Essential Drugs Programme [Internet]. [cited 2017 Nov 13]. Available from: http://www.health.gov.za/index.php/medicine/category/195-essential-drugs-programme-edp.

[38] Zhao K, Xue Li, Wudong Guo, et al. Coming Rapidly of Age: Health Technology Assessment in China [Internet]. Available from: https://globalforum.diaglobal.org/issue/may-2018/coming-rapidly-of-age-health-technology-assessment-in-china/.

[39] HTAin. Health Technology Assessment in India - HTAIn [Internet]. 2018 [cited 2018 Aug 29]. Available from: https://dhr.gov.in/sites/default/files/eNewsletter/img/HTAIn/HTAIn10-01-2017.pdf.

[40] Hofman KJ, McGee S, Chalkidou K, et al. National Health Insurance in South Africa: Relevance of a national priority-setting agency. South African Medical Journal. 2015;105:739.

[41] Prinja S. Personal Communication as part of data extraction for article. 2017.

[42] Chen Y, Banta D, Tang Z. Health technology assessment development in China. International Journal of Technology Assessment in Health Care. 2009;25:202–209.

[43] Department of Health Research, Ministry of Health and Family Welfare, Government of India. Records of the Stakeholders Consultation Meeting held on July 27th, 2017 at 11.30 AM in the Conference Room, Department of Health Research (DHR), Ministry of Health & Family Welfare (MoHFW) under the chairmanship of Mr. V.K. Gauba, JS, DHR, MoHFW in connection with the HTA (Health Technology Assessment) of Intra-Ocular lenses for cataract surgery [Internet]. 2017 [cited 2017 Nov 20]. Available from: http://dhr.gov.in/sites/default/files/Records%20of%20Discussion%20-%20Stakeholder%20meeting%2027th%20July%20final.pdf.

[44] Department of Health Research, Ministry of Health and Family Welfare, Government of India. Stakeholder Registration [Internet]. Available from: http://dhr.gov.in/stakeholder-registration.

[45] Mukherjee A. Fiscal Devolution and Health Financing Reform: Lessons for India from Brazil, China and Mexico. Washington, DC: Center for Global Development .; Report No.: CGD Policy Paper 97. .

[46] International Decision Support Initiative (iDSI). iDSI South South knowledge sharing workshops Johannesburg South Africa 2017. [Internet]. 2017 [cited 2017 Aug 24]. Available from: http://www.idsihealth.org/.

[47] Govender M, Letshokgohla M, Basu D. Health technology assessment - a new initiative in South Africa. South African Medical Journal [Internet]. 100. Available from: http://www.scielo.org.za/scielo.php?script=sci_arttext&pid=S0256-95742010000600004.

[48] International Decision Support Initiative (iDSI). First capacity building for HTA in India workshop kicks off in Delhi [Internet]. 2018 [cited 2018 Aug 29]. Available from: https://www.idsihealth.org/blog/first-capacity-building-for-hta-in-india-workshop-kicks-off-in-delhi/.

[49] iDSI health. First capacity building for HTA in India workshop kicks off in Delhi [Internet]. Available from: https://www.idsihealth.org/blog/first-capacity-building-for-hta-in-india-workshop-kicks-off-in-delhi/.

[50] PGIMER Chandigarh. Welcome to PGIMER Chandigarh: Postgraduate Institute for Medical Education Research. [Internet]. [cited 2017 Nov 30]. Available from: http://www.healtheconomics.pgisph.in/.

[51] University of Stellenbosch. Post basic certificate in health technology assessment Cape Town [Internet]. 2017 [cited 2017 Nov 20]. Available from: http://shortcourses.sun.ac.za/courses/c-10/2017-4319.html.

[52] University of Cape Town. Postgraduate Diploma in Health Economics [Internet]. 2017 [cited 2017 Dec 10]. Available from: http://www.publichealth.uct.ac.za/phfm_postgraduate-diploma-health-economics.

[53] University of Witwatersrand. Health economics Johannesburg [Internet]. [cited 2017 Nov 22]. Available from: https://www.wits.ac.za/course-finder/postgraduate/health/health-economics/.

[54] Blumenthal D, Hsiao W. Lessons from the East — China's Rapidly Evolving Health Care System. New England Journal of Medicine. 2015;372:1281–1285.

[55] Kumar K, Singh A, Kumar S, et al. Socio-Economic Differentials in Impoverishment Effects of Out-of-Pocket Health Expenditure in China and India: Evidence from WHO SAGE. Nugent RA, editor. PLOS ONE. 2015;10:e0135051.

[56] La Forgio G, Nagpal S. Government-Sponsored Health Insurance in India – Are you Covered? Washington: World Bank; 2012.

[57] Jain N, Kumar A, Nandrai S, et al. NSSO 71st Round: Same Data, Multiple Interpretations. Econ. Polit. Wkly. 2015;50:84 – 87.

[58] Section27. Health and Democracy [Internet]. Johannesburg; 2017 [cited 2017 Dec 13]. Available from: http://section27.org.za/2007/06/health-and-democracy/.

[59] Competition Commission South Africa. Health Market Inquiry: Provisional Findings and Recommendations Report [Internet]. 2018 [cited 2018 Aug 22]. Available from: http://www.compcom.co.za/wp-content/uploads/2018/07/Health-Market-Inquiry-1.pdf.
